# Bioavailability of intravenous versus subcutaneous administration of the dual GC-A and GC-B designer natriuretic peptide cenderitide in healthy canines

**DOI:** 10.1186/1471-2210-11-S1-P11

**Published:** 2011-08-01

**Authors:** Guido Boerrigter, Lisa C Costello-Boerrigter, Gail J Harty, John C Burnett

**Affiliations:** 1 Cardiorenal Research Laboratory, Mayo Clinic, Rochester, MN, USA

## Background

Heart failure (HF) remains a therapeutic challenge with high morbidity and mortality and frequent hospitalizations. Activators of guanylyl cyclase (GC) A and/or GC-B such as naturally occurring or designer natriuretic peptides (NPs) are promising candidates for chronic drug therapy; however, parenteral administration is required. Pumps or subcutaneous administration similar to those used for insulin therapy are an attractive means of enabling chronic peptide delivery. Cenderitide (also known as CD-NP) is a Mayo designed chimeric NP consisting of C-type NP with the C-terminus of Dendroaspis NP currently in clinical development for the treatment of HF. Cenderitide is a dual agonist at both GC-A and GC-B and is relatively resistant to enzymatic degradation. This study was designed to assess the bioavailability of cenderitide when given as a subcutaneous (SQ) bolus as compared to an intravenous (IV) bolus.

## Hypothesis

We hypothesized that given cenderitide’s relative resistance to enzymatic degradation it would have good SQ bioavailability as compared to IV administration.

## Methods

Ports were implanted into the femoral artery of four healthy mongrel dogs (weight 21-25 kg). After recovery from surgery dogs underwent two studies on two days with one day in between. On study days, animals were placed into a table top sling and the arterial port was connected via a Huber needle to a pressure gauge to allow measurement of mean arterial pressure (MAP) and heart rate and arterial blood sampling. After baseline measurements and blood sampling, animals received either an IV bolus (10 µg/kg) or, on the other study date, a SQ bolus (40 µg/kg) of cenderitide. Blood samples and MAP measurements were collected for 300 minutes post bolus administration. Two animals started with an IV bolus, the two other with a SQ bolus. Cenderitide levels were measured using a CNP radioimmunoassay that crossreacts with cenderitide. Cyclic GMP was measured by radioimmunoassay. *p<0.05 compared to respective baseline values.

## Results

After IV bolus administration, CNP levels were significantly increased compared to baseline from 0.5’ to 10’ after bolus administration (from 7±2 at baseline to a maximum of 8843±3259 pg/mL after 0.5’). Cyclic GMP levels were increased from 2’ to 90’ after IV bolus (from 10±2 to a maximum of 90±7 pmol/mL after 20’). MAP was significantly decreased from 20’ to 60’ post IV bolus (from baseline 118±6 to a minimum of 92±15 mmHg after 45’). After SQ bolus administration, CNP levels were significantly increased compared to baseline from 10’ to 90’ after bolus administration (from 9±2 to a maximum of 2200±393 pg/mL after 40’). Cyclic GMP levels were increased from 20’ to 180’ after bolus (from 10±3 to a maximum of 94±6 pmol/mL after 60’). MAP was significantly decreased from 35’ to 300’ post bolus (from baseline 124±7 to a minimum of 91±6 mmHg after 300’). Based on the areas under the curve and accounting for dose, bioavailability of cenderitide administered as SQ bolus was estimated to be 72±9% of IV bolus administration.

## Conclusion

Cenderitide, a dual GC-A/GC-B agonist with relatively high resistance to enzymatic degradation, had good bioavailability when administered as a SQ bolus as compared to IV administration. SQ administration was associated with a sustained increase in cenderitide immunoreactivity (up to 90’), activation of cGMP (up to 180’ post bolus) and decrease in MAP (up to 300’). These findings provide important and promising information supporting the further development of cenderitide as a therapeutic for chronic SQ delivery.

